# Satisfaction among Cancer Patients Undergoing Radiotherapy during the COVID-19 Pandemic: An Institutional Experience

**DOI:** 10.3390/curroncol28020142

**Published:** 2021-04-10

**Authors:** Vanessa Di Lalla, Haley Patrick, Nicolas Siriani-Ayoub, John Kildea, Tarek Hijal, Joanne Alfieri

**Affiliations:** Department of Radiation Oncology, McGill University Health Centre, Montreal, QC H4A 3J1, Canada; haley.patrick@mail.mcgill.ca (H.P.); Nicolas.siriani-ayoub@mail.mcgill.ca (N.S.-A.); john.kildea@mcgill.ca (J.K.); tarek.hijal@mcgill.ca (T.H.); joanne.alfieri@mcgill.ca (J.A.)

**Keywords:** COVID-19, patient experience, quality of care, pandemic-based practices, radiation oncology

## Abstract

The COVID-19 pandemic has shifted oncology practices to prioritize patient safety while maintaining necessary treatment delivery. We obtained patient feedback on pandemic-based practices in our radiotherapy department to improve quality of patient care and amend policies as needed. We developed a piloted questionnaire which quantitatively and qualitatively assessed patients’ pandemic-related concerns and satisfaction with specific elements of their care. Adult patients who were treated at our Centre between 23 March and 31 May 2020, had initial consultation via telemedicine, and received at least five outpatient fractions of radiotherapy were invited to complete the survey by telephone or online. Relative frequencies of categorical and ordinal responses were then calculated. Fifty-three (48%) out of 110 eligible patients responded: 32 patients by phone and 21 patients online. Eighteen participants (34%) admitted to feeling anxious about hospital appointments, and only five (9%) reported treatment delays. Forty-eight patients (91%) reported satisfaction with their initial telemedicine appointment. The majority of patients indicated that healthcare workers took appropriate precautions, making them feel safe. Overall, all 53 patients (100%) reported being satisfied with their treatment experience during the pandemic. Patient feedback is needed to provide the highest quality of patient care as we adapt to the current reality.

## 1. Introduction

On 11 March 2020, the World Health Organization declared SARS-CoV-2 and the resulting illness, COVID-19, a pandemic [1]. As of January 2021, over 90 million cases and nearly 1.72 million deaths have been confirmed worldwide [2]. However, the true toll of this pandemic is likely even higher due to the strain on worldwide healthcare systems impacting the health of patients with common illnesses [3]. In particular, cancer patients are at increased risk of death from the virus and virus-based interruptions in care. Early data from China reported that cancer patients with COVID-19 were more likely to suffer severe sequelae compared to those without a cancer diagnosis [4]. This was then confirmed by later international studies [5,6,7]. In addition to deaths related to the virus itself, population-based models predicted that the pandemic would increase cancer deaths in 2020 due to delays in cancer diagnoses, secondary to healthcare systems focusing their efforts on fighting the pandemic [8]. 

At the beginning of the pandemic, Canadian cancer centers looked to the experiences of Chinese and European centers for guidance on best pandemic practices in caring for this vulnerable population. These early recommendations largely focused on the importance of patient and staff screening, masking and disinfection, and physical distancing [9,10,11,12]. Canadian provinces have since created new policies for radiation oncology departments that seek to provide the best possible care while ensuring the safety of patients and staff. These include categorizing patients based on priority to treat in order to reduce oncology’s resource load on other departments, an increased use of telemedicine and hypofractionated treatments to reduce the number of hospital visits for patients [13,14,15]. Moreover, individual centers may enact additional policies like remote check-in services for appointments and additional waiting areas to further reduce opportunities for patient-to-patient transmission [16]. For example, our center chose to offer patients a remote cell phone appointment check-in as well as a designated parking lot where they could wait to be notified to enter the hospital for treatment. The Italian radiotherapy experience has taught us that while some patients feel reassured by some of the measures put in place since the pandemic, many still report fears of being touched or positioned on the treatment table and increased anxiety knowing they must come to hospital for treatment [17]. 

While cancer centers have been operating under these new policies for nearly a year now, little is known about how these policy changes have impacted patient satisfaction with care. To date, most publications addressing or surveying the cancer patient experience during the pandemic are short communications or opinion pieces [17], studies focusing on patient perceptions of the virus [18], or their psychological distress caused by the virus [19,20,21]. A multi-center study conducted in Poland found that in addition to the anxiety surrounding their cancer diagnosis, cancer patients are also anxious about the virus and its impact on their treatments [21]. This anxiety relates to delayed treatments and patient perceived loss of treatment effectiveness. Furthermore, a survey conducted by Cancer Research UK reports a significant reduction in the proportion of patients who rated their care as “very good” [22], but it is unclear as to what components of care respondents were less satisfied with. Given that cancer centers are now treating patients during the second wave of the pandemic, without clear patient feedback on what pandemic practices do and do not work well for them, it is imperative to obtain this feedback as soon as possible in order to be able to amend policies to improve patient care. 

The aim of this study was to determine patient satisfaction with their care during the pandemic in a qualitative and quantitative manner. Additionally, we evaluated patient adoption and satisfaction with our center-specific pandemic safety measures with the intention to amend policies based on patient feedback where possible. 

## 2. Materials and Methods

### 2.1. Study Design and Population

This single-institution descriptive cross-sectional study consisted of a questionnaire distributed to patients treated at the McGill University Health Centre (MUHC) radiation oncology department during the first wave of the COVID-19 pandemic. Ethics approval was granted by the MUHC Research Ethics Board following a full review (project number 2021-7040). As we were interested in the new patient experience for conventional external beam treatments during the initial phase of the global pandemic, eligible patients were outpatients aged 18 years or older who conducted their initial consultation by telemedicine between 23 March and 31 May 2020 and underwent five or more fractions of radiotherapy. This was selected as the minimum number of treatments to ensure respondents had enough experience to develop informed opinions on center operations and to align with more extreme hypofractionated regimens for prostate and breast cancers that were adopted based on international recommendations [9,13,14,15]. 

### 2.2. Survey Development

Since we could not find any existing survey tools in the literature, we designed a novel patient satisfaction survey using an evidence-based approach to assess patient satisfaction [23]. Topics of interest including patient anxiety levels, communication of safety protocols, telemedicine experience, and in-person treatment experience were identified in planning meetings with clinical staff and used to inform survey development. As our center operates bilingually, questions were developed in tandem in English and French and carefully revised by bilingual clinicians and researchers to ensure clarity and plain language in both versions. After initial survey development was complete, a feasibility study was conducted in both languages on a small group of patients by SMS recruitment to assess the expected response rate, formatting and ease of use of the online survey instrument, and content clarity. Information from this feasibility study was used to guide minor revisions in question wording in order to increase clarity for participants.

The finalized survey consisted of 21 quantitative and qualitative questions which included categorical (yes/no/I don’t know) questions, 5-point Likert scale questions (ranging from strongly disagree to strongly agree), and short answer free-text questions. Survey instruments are available as supplementary material. 

### 2.3. Data Collection

Eligible patients were identified using our center’s oncology information system (Aria, Varian Medical Solutions) and contacted by phone for participation. The study purpose and ethical considerations were explained and clarified as needed prior to obtaining verbal consent, as approved by the Research Ethics Board. An emailed copy of the consent form was offered to participants for their records. Participants were then given the option to complete the survey by phone or online in their preferred language. Patients who selected the online option were sent the survey instrument URL and a copy of the consent form by email. Care was taken to ensure the research team member contacting patients was not involved in the patient’s current or future medical care, and this was clearly explained to participants at the time of consent. 

Once eligible patients were identified and charts were reviewed, patient recruitment began in September 2020 and concluded in October 2020. Individuals who did not answer phone calls after three attempts were assumed to decline participation and were not contacted further. 

### 2.4. Statistical Analysis

Relative frequencies of categorical and ordinal responses were calculated. For free-text qualitative data, answers were segmented into individual phrases and collaboratively codified by two researchers using a deductive coding methodology. Phrases were first codified into one of 12 common comment topics identified during the segmentation phase, then codified by commentor attitude towards the topic (positive, negative, or neutral). Chi square tests were used to assess and compare the distribution of commentor attitudes per topic to patient satisfaction scores from other questions. 

Phone and online responses were compared to determine if social desirability bias resulted in phone respondents answering more positively. Mann Whitney U tests were used for comparisons of ordinal data and Chi Square tests for categorical data. Similar comparisons were also performed with patient responses from our initial feasibility study to verify if results would have been impacted had these patients been included in the main study. Statistical significance was defined as α < 0.05. 

## 3. Results

### 3.1. Response Rate and Demographic Characteristics 

We identified 118 eligible patients, who had had their initial consultation by telemedicine between 23 March and 31 May 2020 and underwent radiotherapy at our Centre. During our survey administration, eight patients were deemed ineligible for reasons including death, inability to speak English or French, or the patient reporting that initial consultation was not done via telemedicine. Therefore, 110 patients were ultimately eligible for the study. Out of the eligible patients, 53 patients completed the questionnaire, for a response rate of 48%: 32 patients did so by phone and 21 patients did so online. Patient characteristics are summarized in Table 1. Response rates between the feasibility and main cohorts did not vary significantly except on adoption of phone-check practices (addressed later). 

### 3.2. Pandemic Related Concerns 

**Figure 1:**
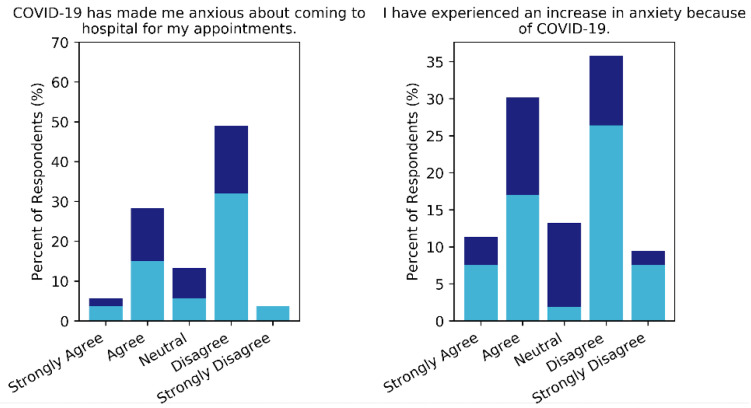
Responses to questions about patient anxiety due to COVID-19. Online responses are shown in indigo and phone responses in sky blue.

Of the 53 respondents, 22 (41.5%) admitted they had experienced some level of increased anxiety due to the pandemic and 18 (34.0%) agreed that COVID-19 had made them anxious about coming to hospital for their appointment (Figure 1). There were no significant differences in rates of reported anxiety between phone and online populations (*p* > 0.2). Of the 18 participants that admitted to feeling anxious about hospital appointments, more than half specified their main concern was contracting the virus because of their appointments and at least two voiced concern over possible treatment delays. Only five patients (9.4%) reported delays in their treatment due to the pandemic, with reasons split between surgery postponement (*n* = 3) and non-urgency of treatment (*n* = 2). Response rates between the phone and online cohorts were different (*p* = 0.02), though this is most likely due to more online participants being unsure of any treatment delays. 

### 3.3. Initial Telemedicine Appointment Experience 

**Figure 2:**
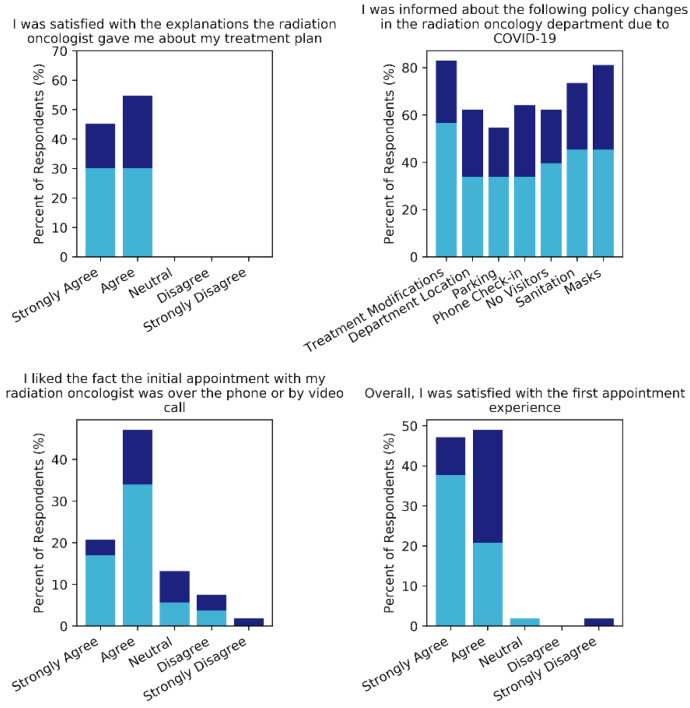
Responses to questions regarding patient experiences during the telemedicine consultation process. Online responses are shown in indigo and phone in sky blue.

When asked about their initial appointment, 100% of respondents either agreed or strongly agreed that their radiation oncologists satisfactorily explained their treatments by telemedicine. All patients but one recalled the policy changes communicated to them at initial appointment, with 55–83% of participants recalling being informed of each individual policy (Figure 2). While 36 patients (67.9%) stated they liked that their initial consultation was conducted by telemedicine, 5 (9.4%) indicated some degree of dissatisfaction with the modality. This dissatisfaction was somewhat more pronounced in the online cohort (*p* = 0.046), a trend that carried over to overall satisfaction with initial appointments. Overall, 48 patients (90.7%) reported satisfaction with their initial appointment, with phone respondents strongly agreeing more frequently than online ones (*p* = 0.012). 

### 3.4. Hospital Visit and Treatment Experience 

Regarding optional safety practices enacted at our center, 24 (45.3%, versus 54.7% informed at consultation) patients reported using the radiotherapy designated parking lot directly outside the department’s entrance and only 11 (20.8%, versus 64.2% informed at consultation) indicated they made use of the remote phone-based check-in for appointments. Opinion on the ease of use was generally positive for both practices, with 20 (83.3%) and 8 (72.7%) patients reporting the parking and remote check-in was easy to use, respectively. Phone check-in adoption was significantly lower compared to the feasibility study population (87.5%, *p* < 0.001) likely due to the feasibility survey being distributed to patients registered for SMS messaging. 

**Figure 3:**
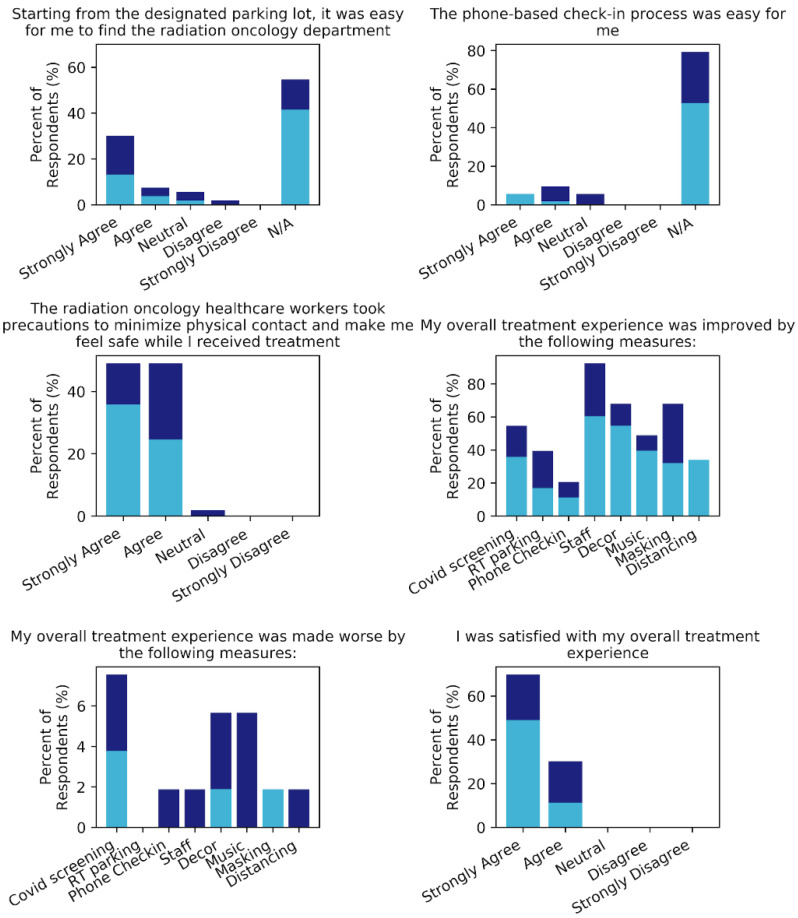
Responses to questions about patient satisfaction with treatment appointments and overall patient experience. Online responses are shown in indigo and phone in sky blue.

When asked about their treatment experiences, all patients but one agreed that the radiation oncology healthcare workers took appropriate precautions to reduce risk of transmission and make them feel safe. Feedback on specific elements that improved patient experiences varied, but factors selected by the majority of patients included: symptom screening practices (54.7%), staff (92.5%), department décor including artwork lining the hallways and treatment rooms (67.9%), and wearing of surgical masks (67.9%). Additionally, 49.0% of patients also indicated that music selected by them and played during their treatment improved their overall experience. While very few patients reported that the new safety measures worsened their treatment experience, the most common complaints were about the COVID-19 screening measures (*n* = 4), décor or music (*n* = 3), and challenges communicating with staff or physicians (*n* = 2). Overall, satisfaction with the overall treatment experience was high, with all 53 patients either agreeing or strongly agreeing with this statement. We did note however, that phone respondents were more likely to strongly agree with this statement than online ones (*p* = 0.03). These results are summarized in Figure 3, and individual responses to selected questions are available as supplementary material (Table S1).

### 3.5. Qualitative Responses 

We collected qualitative data in the form of short answers. Twelve main themes from the patient experience emerged from participant responses, some of which were discussed more positively or negatively than others (Table 2). Patients were overwhelmingly positive about staff attitudes and attentiveness (*p* = 0.004). However, nearly all mentions of communication were negative (*p* = 0.001) and commonly stressed hearing difficulties with masks and telemedicine, or challenges with follow-ups and future appointment scheduling. Comments on general health concerns and other health specialties were also predominantly negative and frequently mentioned fears concerning COVID-19 infection, delayed surgeries, and difficulties with referrals for non-oncologic healthcare. Topics that had more mixed responses included comments such as improvements in pandemic-related anxiety resulting from safety measures, appreciation of the décor but frustration with reduced seating/waiting room crowding, and complaints regarding daily pandemic-screening. A selection of comments is provided in the Supplementary Materials (Table S2). 

Associations between respondent attitudes and overall satisfaction existed for both telemedicine and treatment experiences (Table 2). Patients who reported being “very satisfied” with their treatments were more likely to give positive feedback as opposed to those who reported only being “satisfied” (*p* < 0.001). A similar distribution in positive and negative feedback also existed between “very satisfied” and “satisfied” attitudes towards the overall telemedicine experience (*p* = 0.001), albeit with the addition of some “neutral” and “very dissatisfied” responses. 

## 4. Discussion

In order to assess the impact that pandemic-related changes had on patient experience in our radiotherapy department, we conducted a descriptive cross-sectional study of patients treated during the early pandemic. Overall satisfaction with care was high, with 90.7% of respondents reporting satisfaction with telehealth consults and 100% reporting satisfaction with treatment appointments. Similarly high satisfaction rates were found in an Italian center where 89.6% of patients reported their treatment quality to be good or excellent during the pandemic [24]. While satisfaction rates reported pre-pandemic range from 76.2–95.7% [25,26,27,28], comparison between these results and our own is challenging due to use of different variables in survey instruments, as well as cultural and contextual differences between centers. 

A major change implemented early on in the pandemic is the shift towards telemedicine. Similar satisfaction rates (75–92%) to our 90.7% have been reported by several centers in the US [29,30,31,32], with 70% of patients in one survey stating telemedicine appointments made them feel safer [29]. However, as evidenced in a study by Zimmerman et al., only 23.3% of patients feel telemedicine services can fully replace regular standards of care, with the majority (77.1%) preferring it be used in addition to regular services [31]. In our study, despite overall satisfaction with telemedicine, the most common complaint was poor communication quality (clarity and frequency). While this can be somewhat addressed with more frequent video-based telemedicine, it is unlikely telemedicine can fully replace all in-person appointments, as physicians are concerned about missing important clinical indicators they would otherwise perceive when in-person [29]. 

Overall, the majority of patients were aware of and appreciated new protocols established during the pandemic with few reports of these measures worsening patient experiences. The most disliked protocol was the daily screening questionnaires, which may have been due to respondent fatigue [33]. Increased patient anxiety due to the pandemic was similar to percentages reported in other studies [19,20,21,34], with several patients noting their anxiety reduced after witnessing the protocols at their first treatment appointments. However, while new pandemic protocols did not appear to worsen patient experiences directly, several patient accounts indicate indirect effects are being felt. Reports of reduced access to supportive care, scheduling and communication issues, and difficulties building rapport with staff are suggestive of broader organizational challenges that our center needs to work on to ensure quality is maintained across the full cancer care spectrum. 

To our knowledge, this is the first study conducted assessing patient satisfaction with new pandemic safety precautions at a high-volume radiation oncology center in Canada. Our center sees approximately 4000 new patients per year with over 3000 radiotherapy treatments per year. While our study provides valuable insight into patient attitudes towards departmental pandemic measures, we recognize some inherent limitations exist. Due to the eligibility criteria, sample size was limited to consenting patients who received multi-fraction treatments during the initial pandemic-related service modification period, and further limited by exclusion of those recruited to the feasibility study. While this reduced the power of the study, inclusion of the feasibility group would not have affected the main findings. The retrospective cold-call nature of the study, in addition to possibly introducing recall bias, may have also influenced sample size due to individuals screening their calls and diminished interest in providing feedback over time. The combination of these factors may have led to the exclusion of patients who had greater anxiety and/or less positive experiences than those who participated, such as individuals who chose to forego treatment specifically because of the pandemic. In addition, while question wording bias is possible, we attempted to decrease this by ensuring a balance of questions, including negative and positive responses, avoiding wording that produces emotional responses, diversifying question styles, and including an undecided option. There was also evidence of social desirability bias in the data, as online respondents were less satisfied with elements of care than phone respondents. This occurred despite efforts to mitigate the bias with non-physician surveyors. While a purely online survey may have better addressed this limitation, we were concerned about introducing a selection bias for younger patients who may be more comfortable using technology, due to age being a predictor of patient satisfaction seen in other studies [30,35,36]. Despite these limitations, we believe that our study provides valuable insights into the patient experience during the early pandemic, particularly for areas for future improvement. 

## 5. Conclusions

Unprecedented changes in radiation oncology care practices have been swiftly adopted on a global scale in order to reduce risks of death and hospitalizations in patients and healthcare workers. Although these policy changes may provide sufficient infection control, they may also lead to unintended reductions in quality of care and patient satisfaction that risk becoming commonplace until the pandemic is fully controlled. For this reason, evaluation of and reflection on patient satisfaction with care during this time is essential to ensure that the patient remains the center of our focus [16]. Our study highlights that patients have positively responded to pandemic-related policies, however minor challenges in communication remain. 

## Figures and Tables

**Table 1:** Patient Demographic Data.

		Total (*n* = 53)
Age	18–25	0
	26–45	3 (5.66%)
	46–65	16 (30.19%)
	66–75	25 (47.17%)
	Over 75	9 (16.80%)
Gender	Male	17 (32.08%)
	Female	36 (67.92%)
Cancer Diagnosis	Breast	24 (45.28%)
	Prostate	3 (5.66%)
	Lung	7 (13.21%)
	Colon	3 (5.66%)
	Gynecologic	1 (1.89%)
	Other	15 (28.30%)
Diagnosis Date	Prior to March 2020	30 (56.60%)
	March 2020 and later	22 (41.51%)
	Unknown	1 (1.89%)
Cancer treatments received	Radiotherapy	53 (100.00%)
	Systemic Therapy	34 (64.15%)
	Surgery	35 (66.04%)

**Table 2:** Qualitative data from survey responses, codified by commentary topic and attitude. Associations between respondent attitudes to free-text qualitative questions and quantitative experience scores are also presented.

Frequency of Commentary on Various Aspects of Treatment Experience				
	Total	Positive Attitude	Negative Attitude	*p*-Value
Communication	14	1	13	0.001
Staff	12	11	1	0.004
Clinic Organization	7	2	5	ns
Environment & Decor	7	4	3	ns
Pandemic Safety Procedures	10	5	5	ns
Treatment Procedures	6	0	4	ns
Health Concerns (General)	12	0	11	0.001
Emotional State	7	4	3	ns
Waiting	3	2	1	ns
Transit to/from Centre	5	3	2	ns
Other Specialists	5	0	5	0.025
Generic Sentiments	14	14	0	<0.001
Relation to overall treatment experience satisfaction *p* < 0.001				
	Very Satisfied	Satisfied		
Positive Attitude	40	6		
Negative Attitude	29	24		
Relation to overall telehealth consult experience satisfaction *p* = 0.001				
	Very Satisfied	Satisfied	Neutral	Very Unsatisfied
Positive Attitude	37	6	0	3
Negative Attitude	23	26	1	3

## Data Availability

Data is contained within the article or supplementary material.

## Supplementary Materials

The following are available online at https://www.mdpi.com/article/10.3390/curroncol28020142/s1, Table S1: Individual responses to selected questions; Table S2: Qualitative data from survey responses; Patient surveys (available in English and French).
