# Supplementary material for: Satisfaction among Cancer Patients Undergoing Radiotherapy during the COVID-19 Pandemic: An Institutional Experience
Source: Curr Oncol. 2021 Apr 10;28(2):1507–17. doi: 10.3390/curroncol28020142 (PMC8167586; doi:10.3390/curroncol28020142)
Supplement: Supplementary file 1 [file curroncol-28-00142-s001.pdf]

Supplemental Materials

In the following document, you will find:

1) **Supplementary Tables**
   Supplementary Table 1: Individual responses to selected questions…………………………………..page 2
   Supplementary Table 2: Qualitative data from survey responses……………………………………….page 3

2) **Patient questionnaires administered during the study, available in English and French**
   English version……page 4
   French version……page 7

Category	Question	Response	Phone Responses n=32 (%)	Online Responses n=21 (%)
**COVID-19 related anxiety**	*I have experienced an increase in anxiety because of COVID-19.*	Strongly agree	4 (12.5)	2 (9.5)
		Agree	9 (28.1)	7 (33.3)
		Neutral	1 (3.1)	6 (28.6)
		Disagree	14 (43.8)	5 (23.8)
		Strongly disagree	4 (12.5)	1 (4.8)
	COVID-19 has made me anxious about coming to hospital for my appointments.	Strongly agree	2 (6.3)	1 (4.8)
		Agree	8 (25.0)	7 (33.3)
		Neutral	3 (9.4)	4 (19.0)
		Disagree	17 (53.1)	9 (42.9)
		Strongly disagree	2 (6.3)	0 (0)
**Initial Telemedicine Experience**	*I liked the fact the initial appointment with my radiation oncologist was over the phone*	Strongly agree	9 (28.1)	2 (9.5)
		Agree	18 (56.3)	7 (33.3)
		Neutral	3 (9.4)	4 (19.0)
		Disagree	2 (6.3)	2 (9.5)
		Strongly disagree	0 (0)	1 (4.8)
	Overall, I was satisfied with the first appointment experience	Strongly agree	20 (62.5)	5 (23.8)
		Agree	11 (34.4)	15 (71.4)
		Neutral	1 (3.1)	0 (0)
		Disagree	0 (0)	0 (0)
		Strongly disagree	0 (0)	1 (4.8)
**Hospital Treatment Experience**	*The radiation oncology healthcare workers took precautions to minimize physical contact and make me feel safe*	Strongly agree	19 (59.4)	7 (33.3)
		Agree	13 (40.6)	13 (61.9)
		Neutral	0 (0)	1 (4.8)
		Disagree	0 (0)	0 (0)
		Strongly disagree	0 (0)	0 (0)
	I was satisfied with my overall treatment experience	Strongly agree	26 (81.3)	11 (52.4)
		Agree	6 (18.8)	10 (47.6)
		Neutral	0 (0)	0 (0)
		Disagree	0 (0)	0 (0)
		Strongly disagree	0 (0)	0 (0)

Table S1: Individual responses to selected questions

Topic	Patient Responses
**COVID-19 related anxiety**	*"I was anxious at first but then found the hospital is a pretty safe place. Everyone "gets it""*<br><br>*"Even though the hospital followed all the recommended health guidelines, for which I was truly grateful, I was always concerned about picking up the virus from anyone in passing."*<br><br>*"My reduced immune system makes me vulnerable to infection."*<br><br>*"Anxious at first, but felt comfortable after 2 weeks of treatment"*
**Initial appointment via telemedicine**	*"Phone very thorough, more convenient, could wait at home with less risk"*<br><br>*"Spend more time explaining treatment results, next steps etc. and give more opportunity for questions because it is difficult to understand the explanations when not conducting the appointment in person (facial and body-language is absent)."*<br><br>*"Very well organised."*<br><br>*"Difficult to hear over phone sometimes"*
**Hospital visits and treatment experience**	*"It was an amazing experience for me, and I am so grateful for the wonderful staff that took care of me, including the phone calls and kindness and understanding of everyone."*<br><br>*"Treatment was excellent, staff courteous and polite."*<br><br>*"Always felt safe and comfortable, staff are very kind and professional"*<br><br>*"There is always a wait time in the waiting room, but in reality, it is not that long" (translated from French)*

Table S2: Qualitative data from survey responses

2) Survey Questions – English

Part A: Demographic Information

This section focuses on questions about you as a person that can help determine if your experience was similar to other patients that are similar to you. Please give as much information as you are comfortable giving.

1. Age:
    - 18-25
    - 26-45
    - 46-65
    - 66-75
    - Over 75
2. Patient gender
    - Male, Female, <u>or</u> Prefer not to answer
3. Cancer diagnosis:
    - Breast, prostate, lung, colon, bladder, gynecological (uterus, cervical, ovarian), head and neck, <u>or</u> other: please specify
4. Date of cancer diagnosis (year and month, if possible):
    - Date format free answer
5. Cancer treatments you have received (select all that apply):
    - Chemotherapy
    - Radiation Therapy
    - Surgery
    - Immunotherapy
    - Other (please specify)

Part B: Questions relating to COVID-19

This section focuses on how the pandemic has affected your attitude towards receiving radiotherapy treatments.

6. I have experienced an increase in anxiety because of the pandemic:
    - 0 strongly agree
    - 1 agree
    - 2 neither agree nor disagree
    - 3 disagree
    - 4 strongly disagree
7. The pandemic has made me anxious about coming to hospital for my appointments
    - 0 strongly agree
    - 1 agree
    - 2 neither agree nor disagree
    - 3 disagree
    - 4 strongly disagree
8. If you answered "Agree" or "Strongly agree" to the previous question please tell us why
    - Free text response
9. My cancer treatments were/have been postponed because of COVID-19.
    - Yes, No, or I don't know
10. If you answered "Yes" to the previous question please tell us why:
    - I tested positive for COVID-19
    - I had been in close contact with someone who tested positive for the virus
    - I was afraid of coming to the hospital

- I had another illness that prevented me from going to the hospital
- My doctor told me my treatment was not urgent and could wait
- Other: please specify

Part C: Questions on Your Initial Appointment

This section focuses on your experience during your first appointment with your radiation oncologist (the doctor designing your radiotherapy treatment). Please indicate your level of agreement with the following statements.

11. I liked the fact that the initial appointment with my radiation oncologist was held over phone or video call:
    - 0 strongly agree
    - 1 agree
    - 2 neither agree nor disagree
    - 3 disagree
    - 4 strongly disagree
    - 5 Not applicable
12. I was informed about the following policy changes in the radiation oncology department due to COVID-19 (check all that apply):
    - How my treatment process would work
    - How to get to the radiation oncology department
    - The "no visitor" policy during the pandemic
    - What sanitation measures are in place to protect me and staff
    - I am recommended to wear a mask while in the hospital
    - I have the option to check in for appointments with my mobile phone
    - There is a designated parking lot just for radiation oncology patients
    - Other (please specify)
13. I was satisfied with the explanations the physician gave me about my treatment plan:
    - 0 strongly agree
    - 1 agree
    - 2 neither agree nor disagree
    - 3 disagree
    - 4 strongly disagree
14. Overall, I was satisfied with the first appointment experience:
    - 0 strongly agree
    - 1 agree
    - 2 neither agree nor disagree
    - 3 disagree
    - 4 strongly disagree
15. Do you have any comments or concerns on how we can improve phone and video call appointments? If so, please specify:
    - Free text response

Part D: Hospital Visit and Treatment

16. From the designated parking lot, it was easy for me to find the radiation oncology department:
    - 0 strongly agree
    - 1 agree
    - 2 neither agree nor disagree
    - 3 disagree
    - 4 strongly disagree

- 5 I did not use the parking lot

17. The phone-based check-in process was easy for me:
    - 0 strongly agree
    - 1 agree
    - 2 neither agree nor disagree
    - 3 disagree
    - 4 strongly disagree
    - 5 I did not use the phone-based check-in
18. If you answered "disagree" or "strongly disagree" to either the parking lot or the phone check-in questions, please tell us why:
    - Free text response
19. The radiation oncology healthcare workers took precautions to minimize physical contact and make me feel safe:
    - 0 strongly agree
    - 1 agree
    - 2 neither agree nor disagree
    - 3 disagree
    - 4 strongly disagree
20. My overall treatment experience was improved by the following measures (check all that apply):
    - COVID-19 symptom screening by phone and at the hospital entrance
    - The designated parking lot
    - The optional phone-based check-in process
    - Staff wearing masks and increasing cleaning and disinfecting measures
    - Staff attitudes (comforting, helpful, etc)
    - The décor of the radiotherapy suites
    - Music being played during my treatment
    - Other (please specify)
21. My overall treatment experience was made worse by the following measures (check all that apply):
    - COVID-19 symptom screening by phone and at the hospital entrance
    - The designated parking lot
    - The optional phone-based check-in process
    - Staff wearing masks and increasing cleaning and disinfecting measures
    - Staff attitudes (comforting, helpful, etc)
    - The décor of the radiotherapy suites
    - Music being played during my treatment
    - Other (please specify)
22. I was satisfied with my overall treatment experience:
    - 0 strongly agree
    - 1 agree
    - 2 neither agree nor disagree
    - 3 disagree
    - 4 strongly disagree
23. Do you have any other comments or concerns about your treatment experience you would like to voice at this time (please specify)?
    - Free text response

Survey Questions - French

Partie A : Questions démographiques
Cette section se concentre sur des questions de vous en tant que personne qui peuvent aider à déterminer si votre expérience était similaire à celle d'autres patients qui vous ressemblent. Veuillez donner autant d'informations que vous le souhaitez.

1. Âge du patient
   - 18-25
   - 26-45
   - 46-65
   - 66-75
   - Plus de 75 ans
2. Sexe du patient
   - Homme, Femme, <u>ou</u> Je préfère ne pas répondre
3. Diagnostic du cancer
   - Sein, Prostate, Poumon, Colon, Vessie, Gynécologique (utérus, ovarien, cervical), Tête et cou , <u>ou</u> Autre (spécifiez)
4. Date du diagnostic de cancer (année et mois, si possible)
   - Date libre
5. Traitement(s) reçu(s) : plus d'une réponse possible
   - Chimiothérapie
   - Radiothérapie
   - Chirurgie
   - Immunothérapie
   - Autre (spécifiez)

Partie B : Questions à propos de la pandémie COVID-19
Cette section se concentre sur la façon dont la pandémie a affecté votre attitude à l'égard des traitements de radiothérapie. Veuillez indiquer votre niveau d'accord avec les déclarations suivantes.

6. J'ai ressenti plus d'anxiété à cause de COVID-19:
   - 0 fortement d'accord
   - 1 d'accord
   - 2 neutre
   - 3 désaccord
   - 4 fortement en désaccord
7. COVID-19 m'a rendu anxieux/anxieuse de venir à l'hôpital pour mes rendez-vous
   - 0 fortement d'accord
   - 1 d'accord
   - 2 neutre
   - 3 désaccord
   - 4 fortement en désaccord
8. Si vous avez répondu "D'accord" ou "Fortement d'accord" à la question précédente, veuillez nous expliquer pourquoi:
   - Texte libre
9. Mes traitements contre le cancer ont été reportés en raison de COVID-19
   - Oui, Non, <u>ou</u> Je ne sais pas
10. Si oui, SVP dîtes-nous pourquoi :
    - J'ai été testé positif au COVID-19

- J'avais été en contact étroit avec quelqu'un qui avait été testé positif au COVID-19
- J'avais peur de venir à l'hôpital
- J'ai eu une autre maladie qui m'a empêchée d'aller à l'hôpital
- Mon médecin m'a dit que mon traitement n'était pas urgent et pouvait attendre
- Autre (spécifiez)

Partie C : Questions sur le rendez-vous initial

Cette section se concentre sur votre expérience lors de votre premier rendez-vous avec votre radio-oncologue (le médecin qui conçoit votre traitement de radiothérapie). Veuillez indiquer votre niveau d'accord avec les déclarations suivantes.

11. J'ai apprécié le fait que le rendez-vous initial avec mon radio-oncologue ait eu lieu par téléphone ou par appel vidéo :
    - 0 fortement d'accord
    - 1 d'accord
    - 2 neutre
    - 3 désaccord
    - 4 fortement en désaccord
    - 5 N'est pas applicable
12. J'ai été informé des changements suivants dans le département de radio-oncologie en raison de COVID-19 (cochez toutes les réponses qui s'appliquent) :
    - Comment mon processus de traitement fonctionnerait
    - Comment se rendre au département de radio-oncologie
    - La politique du «aucun visiteurs» pendant la pandémie
    - Quelles mesures d'assainissement sont en place pour me protéger et protéger le personnel
    - Il est recommandé de porter un masque à l'hôpital
    - Je peux vérifier les rendez-vous à distance à l'aide de mon téléphone si je veux
    - Il y a un stationnement réservé aux patients en radio-oncologie
    - Autre (spécifiez)
13. Je suis satisfait des explications que le radio-oncologue m'a données concernant mon plan de traitement:
    - 0 fortement d'accord
    - 1 d'accord
    - 2 neutre
    - 3 désaccord
    - 4 fortement en désaccord
14. Globalement, je suis satisfait de mon expérience de rendez-vous initial:
    - 0 fortement d'accord
    - 1 d'accord
    - 2 neutre
    - 3 désaccord
    - 4 fortement en désaccord
15. Commentaires sur la façon dont nous pouvons améliorer nos rendez-vous par téléphone ou appel vidéo (SVP spécifiez) :
    - Texte libre

Partie D : Questions sur les visites à l'hôpital pour traitement

Cette section se concentre sur votre expérience lors de la réception de vos traitements de radiothérapie. Veuillez indiquer votre niveau d'accord avec les déclarations suivantes.

16. Venant du stationnement désigné, il m'a été facile d'accéder le département de radio-oncologie :
    - 0 fortement d'accord
    - 1 d'accord
    - 2 neutre
    - 3 désaccord
    - 4 fortement en désaccord
    - 5 Je n'ai pas utilisé le stationnement désigné
17. Le processus d'enregistrement à mes rendez-vous par téléphone a été facile pour moi:
    - 0 fortement d'accord
    - 1 d'accord
    - 2 neutre
    - 3 désaccord
    - 4 fortement en désaccord
    - 5 Je n'ai pas utilisé le système d'enregistrement par téléphone
18. Si vous avez répondu «en désaccord» ou «fortement en désaccord» aux questions relatives à l'enregistrement par téléphone ou stationnement, veuillez nous expliquer pourquoi
    - Texte libre
19. Les travailleurs de la sante en radio-oncologie ont pris des précautions pour minimiser les contacts physiques et me faire sentir en sécurité :
    - 0 fortement d'accord
    - 1 d'accord
    - 2 neutre
    - 3 désaccord
    - 4 fortement en désaccord
20. Mon expérience globale de traitement a été améliorée par les mesures suivantes (cochez toutes les réponses qui s'appliquent):
    - Dépistage des symptômes du COVID-19 par téléphone et à l'entrée de l'hôpital
    - Le stationnement désigné
    - Le processus d'enregistrement par téléphone
    - Personnel portant des masques et renforcement des mesures d'assainissement
    - Attitudes du personnel (réconfortant, utile, etc.)
    - Le décor des salles de traitement de radiothérapie
    - Musique jouée pendant mon traitement
    - Autre (spécifiez)
21. Mon expérience globale de traitement a été aggravé par les mesures suivantes (cochez toutes les réponses qui s'appliquent) :
    - Dépistage des symptômes du COVID-19 par téléphone et à l'entrée de l'hôpital
    - Le stationnement désigné
    - Le processus d'enregistrement par téléphone
    - Personnel portant des masques et renforcement des mesures d'assainissement
    - Attitudes du personnel (pas réconfortant, peu serviable, etc.)
    - Le décor des salles de traitement de radiothérapie
    - Musique jouée pendant mon traitement
    - Autre (spécifiez)
22. Je suis globalement satisfait de mon expérience de traitement:
    - 0 fortement d'accord

- 1 d’accord
- 2 neutre
- 3 désaccord
- 4 fortement en désaccord

23. Avez-vous d’autres commentaires concernant votre expérience de traitement (SVP spécifiez)?
